# 4D-DIA proteomic analysis of human placenta in fetal intrauterine growth restriction

**DOI:** 10.3389/fmed.2026.1842267

**Published:** 2026-07-29

**Authors:** Yuanyuan Dang, Xiaoxiao Kong, Tian Yang, Qiuhong Yu, Ping Chen, Mingli Lyu

**Affiliations:** 1 Department of Ultrasound Diagnosis, The International Peace Maternity and Child Health Hospital, School of Medicine, Shanghai Jiaotong University, Shanghai, China; 2 Shanghai Key Laboratory of Embryo Original Diseases, Shanghai, China; 3 Institute of Birth Defects and Rare Diseases, School of Medicine, Shanghai Jiaotong University, Shanghai, China

**Keywords:** ELISA, FGR, human, placenta, proteomic analysis

## Abstract

**Introdution:**

Approximately 60% of fetal intrauterine growth restriction (FGR) cases result from placental dysfunction. 4D-DIA proteomic chips can efficiently identify differentially expressed proteins (DEPs), to explore FGR pathophysiology mechanism caused by placenta dysfuntion.

**Methods:**

This study aimed to identify differentially expressed proteins (DEPs) in placentas from 10 FGR and 10 normal participants using 4D-DIA proteomic chips, by comparing fetal- and maternal-side placental tissues in both groups. GO enrichment and KEGG pathway analyses were applied to interpret the biological functions of DEPs, and ELISA was used to validate candidate proteins in an additional 8 FGR and 10 normal placentas.

**Results:**

In total, 249 DEPs were identified between maternal and fetal sides in the control group (CON-M vs CON-F), whereas only 158 DEPs were found in the FGR group (FGR-M vs FGR-F). Between-group comparisons of the same placental side showed 278 DEPs in FGR-F vs CON-F and 157 DEPs in FGR-M vs CON-M. Candidate proteins included PRPF38A(down-regulated), PRPF38B(up-regulated), THOC2(down-regulated) which associate with mRNA splicing via spliceosome and COX6C(down-regulated), COX7B(up-regulated) which participanting in oxidative phosphorylation).

**Discussion:**

The fetal placental compartment may be the primary contributor to placental dysfunction, and these proteins may be crucial in FGR pathophysiology, requiring further research.

## Introduction

Fetal growth restriction (FGR) means that a fetus fails to achieve its biologically determined growth potential. Number studies have showed that FGR is the main cause for stillbirth or prenatal mortality and morbidity. Even in survives cases, it may face an increased risk of prematurity, adverse underdevelopment outcome and a higher likelihood of developing non-communicable diseased in adulthood (1, 2). Fetuses with a birth weight below the 3rd percentile are at the highest risk of stillbirth and perinatal mortality (3, 4), so the fetal AC or EFW below the 3rd percentile for given growth chart can be used as an isolated diagnostic criterion for FGR at any gestational stage (1).

The pathogenesis of FGR is still unclear. Numerous studies thought it is multifactorial, but mainly relates to impaired placental function which fails to provide enough oxygen and nutrient (4, 5). A better understanding of alterations in placental structural and/or function may reveal the underlying pathology leading of FGR, and help to early diagnosis and therapeutic intervention.

Data-independent acquisition (DIA) technology is a mass spectrometry (MS)-based proteomics technologies which can thoroughly and extremely sensitively detect and quantify proteins in proteomics samples and has higher data acquisition efficiency, reproducibility and quantitative accuracy compared to conventional protein chip system (6, 7). Four-dimensional (4D) proteomics demonstrates greater scanning speed and detection sensitivity. By integrating the advantages of both 4D proteomics and DIA technology, 4D-DIA can significantly enhance data integrity, detection sensitivity and proteomic depth (7, 8).

In order to find potential biomarkers and underlying mechanism associated with FGR, we conducted a proteomic analysis on both fetal and maternal side of placenta tissues from FGR and controls patients using 4D-DIA technology. Then we quantitatively validated these differentially expressed proteins (DEPs) using enzyme-linked immunosorbent assays (ELASA). A comprehensive investigation of these DEPs will hopefully discovery novel biomarkers for FGR diagnosis, provide insights into its pathogenesis and offer crucial direction and decision-making support for prevention and potential reversal of FGR.

## Methods

### Patients

This study was a single center study approved by the Ethics Committee of the International Peace Maternal and Child Health Hospital (IPMCH), Shanghai Jiaotong University (No. GKLW-A-2024-067-01). Participants were recruited from women who underwent prenatal examination and delivered at the IPMCH from January 2022 to July 2022. Informed consents were provided by all participants. A total of 18 FGR patients and 20 women with normal delivered were enrolled. FGR was diagnosed as birth weight below the 3rd percentile of their gestation week (1). Women in the control group should be term deliveries and birth weight above 10rd percentile of their gestation age. Exclusion criteria for both two groups included maternal hypertension (GH), gestational diabetes mellitus (GDM), other pregnancy complications, mother underlying medical conditions, fetal structural abnormalities or chromosomal abnormalities.

Both fetal and maternal side of the placenta villous tissues were collected after delivered, no matter vaginal birth or cesarean section following strict clinical protocols. Sample were stored at −80 °C for subsequent proteomic analysis and ELISA confirmation.

### Sample collection

We have standard operating procedure for placental tissue sampling to clarify the tissue types collected from the maternal and fetal placental surfaces. For sampling on the maternal placental surface (the side without umbilical cord attachment): Three separate tissue blocks were collected, each measuring less than 0.5 cm × 0.5 cm × 0.5 cm. All sampling regions were positioned at least 2 cm from the placental edge and 1.5 cm from the umbilical cord insertion site. Regions showing prominent infarction, thrombosis or calcification were excluded from sampling. Curved scissors were used to cut through the full thickness of the placental tissue block along the periphery. Connective tissue and decidual tissue attached to both sides of the tissue were carefully resected, and excess blood was wiped off with sterile medical gauze. The full-thickness tissue block was then equally divided into two halves: the upper half adjacent to the maternal surface (maternal side) and the lower half adjacent to the fetal surface (fetal side). After rinsing with PBS, each half was cut into small pieces (< 1 cm × 1 cm × 1 cm). Blood vessels and residual connective tissue were further carefully removed, excess liquid was absorbed with sterile gauze, and the tissue pieces were snap-frozen in liquid nitrogen immediately.

Because decidual tissue was completely stripped and discarded from maternal-side specimens, and the outer fetal membranes were fully removed from fetal-side specimens. Consequently, tissues harvested from both the maternal and fetal sides of the placenta consisted predominantly of chorionic villi, with negligible decidual or membranous contaminants.

### Proteomic analysis

A total of 10 samples from FGR group and 10 samples from normal group were randomly selected from IPMCH hospital specimen repository. Each sample includes fetal and maternal placental side for proteomic analysis.

### Sample preparation

A total of 1 mg of each placental villous sample were ground into a powder in liquid nitrogen and homogenized in extraction buffer (4% SDS, 1 mM DTT, 150 mM Tris-HCI, PH 8.0). Protein extraction was carried out by cell lysis at 95 °C for 5 min, followed by sonication on ice. Then the crude extract was incubated at 95 °C again and centrifugated at 14, 000 g for 30 min at 15 °C. The supernatant was collected and the protein concentration was measured using a BCA protein assay kit (Beyotime, China). Appropriate amount of protein solution (200 ~ 400 μg) was taken from each sample and pooled to construct a spectral library. 20 μg protein solution from both the pool sample and the original sample were performed SDS-PAGE electrophoresis test and evaluate the consistency between samples.

### Filter-aided sample preparation (FASP) and digestion

Protein digestion was performed as the FAST protocol for both the original and pool sample (9). UV light spectral density at 280 nm was used to determine the peptide concentration. The 100 μg Pool mixed peptide was classified by HPRP method, and collect all components. All the peptides were desalted with a C18 cartridge (Evosep, Denmark), reconstituted in 40 μL of a 0.1% formic acid solution and estimated the peptide content with OD280. Afterwards 2 μg peptides were took out, and appropriate amount of iRT standard peptides were added. The mixture was then underwent DDA mass spectrometry detection for 60 min.

### DIA mass spectrometry analysis

DIA mass spectrometry analysis was carried out strictly in accordance with the manufacturer’s requirements, which was similar to that described in the literature (7). About 200 ng peptide segments were extracted from each sample and DIA mass spectrometry was performed for 60 min. The UPLC Brucker NanoElute system is used for chromatographic separation. Buffer solution A consisted of 0.1% formic acid in aqueous solution and buffer B solution was composed of 0.1% formic acid acetonitrile solution with a final acetonitrile concentration of 100%. The column was balanced with 100% buffer solution A. Peptides were separated on a 250nmx75 μm analytical column (AURORA UPLC Column) with 1.6 μm-lmC18 beads with autosampler. Flow velocity was 300 nL/min. The liquid phase separation gradient was as follows: from 0 to 45 min, the linear gradient of buffer B solution was from 2 to 22%; from 45 min to 50 min, from 22 to 33%; 50 min to 55 min, from 35 to 80%; 55 min to 60 min, the linear gradient of buffer B solution was maintained at 80%. The samples separated form UPLC Brucker NanoElute system was underwent DIA mass spectrometry using times TOF Pro mass analyzer (Brucker) for 60 min. Primary mass spectrum scan rang: 100–1700 m/z, 1/K0 Start: 0.75 *Vs*/cm^2^, 1/K0 End: 1.40 *Vs*/cm^2^; Intensity Threshold: 5000.00; Absolute Threshold: 10; Target Intensity: 10000 cts/s, Release after: 0.50 min, No. of PASEF MS/MS scans: 10. MS2 used DIA data collection mode. Resolution ratio of mass spectrum: 30,000 (m/z 200), AGC target: 3e6, Maximum IT: auto, MS2 Activation Type: HCD, Normalized collision energy: 30, Spectral data type: profile. Intensity Threshold: 5000.00; Absolute Threshold: 10; Target Intensity: 10000 cts/s, Release after: 0.50 min, No. of PASEFMS/MS scans: 10.

### Bioinformatics analysis

**Figure 1:**
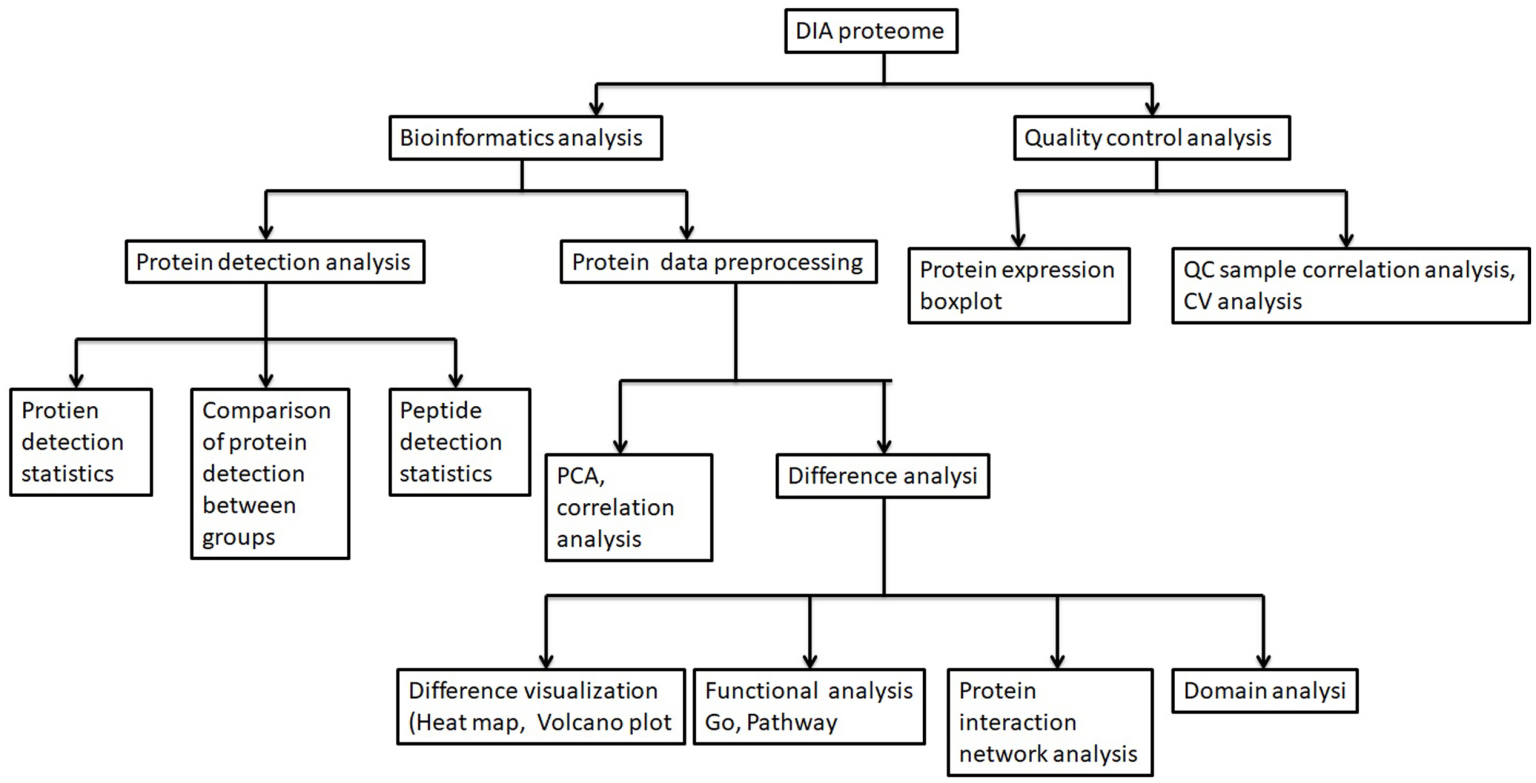
Bioinformation analysis flow chat.

Then DIA-NN search database was employed to conduct a comprehensive bioinformatics analysis. Blast2GO was utilized to annotated the molecular functions (MF), biological processes (BP), and cellular components (CC) of the DEPs (10). Additionally, DEPs were annotated using the Kyoto Encyclopedia of Genes and Geneomes (KEGG) pathway database. Interpro database for functional domain annotation was used to analysis of DEPs. Fisher’s exact test was conducted to determine the significantly enriched Gene Ontology (GO) terms, KEGG pathways, and functional domains. For cluster analysis, quantitative data of the target protein were first normalized. Subsequently, both sample and protein expression dimensions were classified using matplotlib software (V3.7.2), and a hierarchical clustering heatmap was generated. The results were presented in the form of a Venn diagram. Interaction networks of DEPs were generated using the SRING database and visualized with Cytoscape software (V3.9.1). The overall workflow diagram of data analysis is showed in Figure 1.

### Targeted DEPs validation by ELISA

Seven selected candidate biomarker, THO complex subunit 2 (THOC2), Pre-mRNA-splicing factor 38B (PRPF38B), Cytochrome c oxidase subunit 6C (COX6C), Cytochrome c oxidase subunit 7B (COX7B), NADH dehydrogenase [ubiquinone] 1 beta subcomplex subunit 9 (NDUFB9), DNA replication licensing factor MCM5 (MCM5) were evaluated using 18 newly placental samples (8 FGR cases and 10 controls) using commercially available ELAISA kits from Shanghai Jingkang Biological Engineering Co., Ltd. All the tests were performed according the manufacturer’s instructions. The optical density value of each wells were measured using a microtiter plate reader. The protein concentration in each sample was calculated by combining it with the standard curve.

### Statistical analysis

SPSS 24.0 statistical software were used for statistical description. Statistical results were presented as mean ± standard deviation (x̄ ± s). Comparisons between groups were conducted using the independent-sample *t*-test. *p* < 0.05 was considered statistically significant.

## Results

### Clinical characters of patients

All participants were singleton pregnancies. All the participants had no smoking history, heavy alcohol consumption, or drug abuse. No significant differences were observed between two groups in terms of maternal age, BMI, GH, GDM, history of previous pregnancy, if with oligohydramnios and fetal gender. The gestational ages at delivery were significant earlier, and birth weights of infants were significant lower in FGR group compared to the Control group both in 4D-DIA proteomic analysis and ELISA validation cohort (Table 1). Only placentas from the FGR group underwent pathological examination.

**Table 1:** Clinical characters of FGR group and control group.

Group	4D-DIA			ELASA		
	FGR (*N* = 10)	Normal (*N* = 10)	*p*	FGR (*N* = 10)	Normal (*N* = 10)	*p*
Age	33.90 ± 5.93	32.10 ± 2.64	0.07	32.60 ± 2.12	30.75 ± 2.87	0.13
BMI	20.56 ± 2.33	21.78 ± 2.72	0.64	21.14 ± 3.57	20.03 ± 1.60	0.43
GH	0%(0/10)	0%(0/10)	1.00	0%(0/10)	0%(0/8)	1.00
GDM	0%(0/10)	0%(0/10)	1.00	0%(0/10)	0%(0/8)	1.00
First pregnancy	60%(6/10)	50%(5/10)	0.65	50%(5/10)	62.50%(5/8)	0.66
First delivery	70%(7/10)	50%(5/10)	0.36	80%(8/10)	50%(4/8)	0.32
Amniotic fluid^*^	10%(1/10)	0%(0/10)	0.31	10%(1/10)	12.50%(1/8)	1.00
Gender	40%(4/10)	60%(6/10)	0.37	60%(6/10)	12.5%(1/8)	0.07
Delivery age	35.90 ± 1.98	38.49 ± 0.84	0.00	35.56 ± 2.35	38.15 ± 0.93	0.01
Birth Weight	2002.80 ± 348.10	3147.50 ± 461.15	0.00	1939.50 ± 440.41	3244.38 ± 433.96	0.00
Weight of placenta	363.70 ± 94.31			350.10 ± 97.96		

*Amniotic fluid: if accompanied with oligohydramnios fluid before delivery (AFI < 80).

### Quality control and repeatability testing

**Figure 2:**
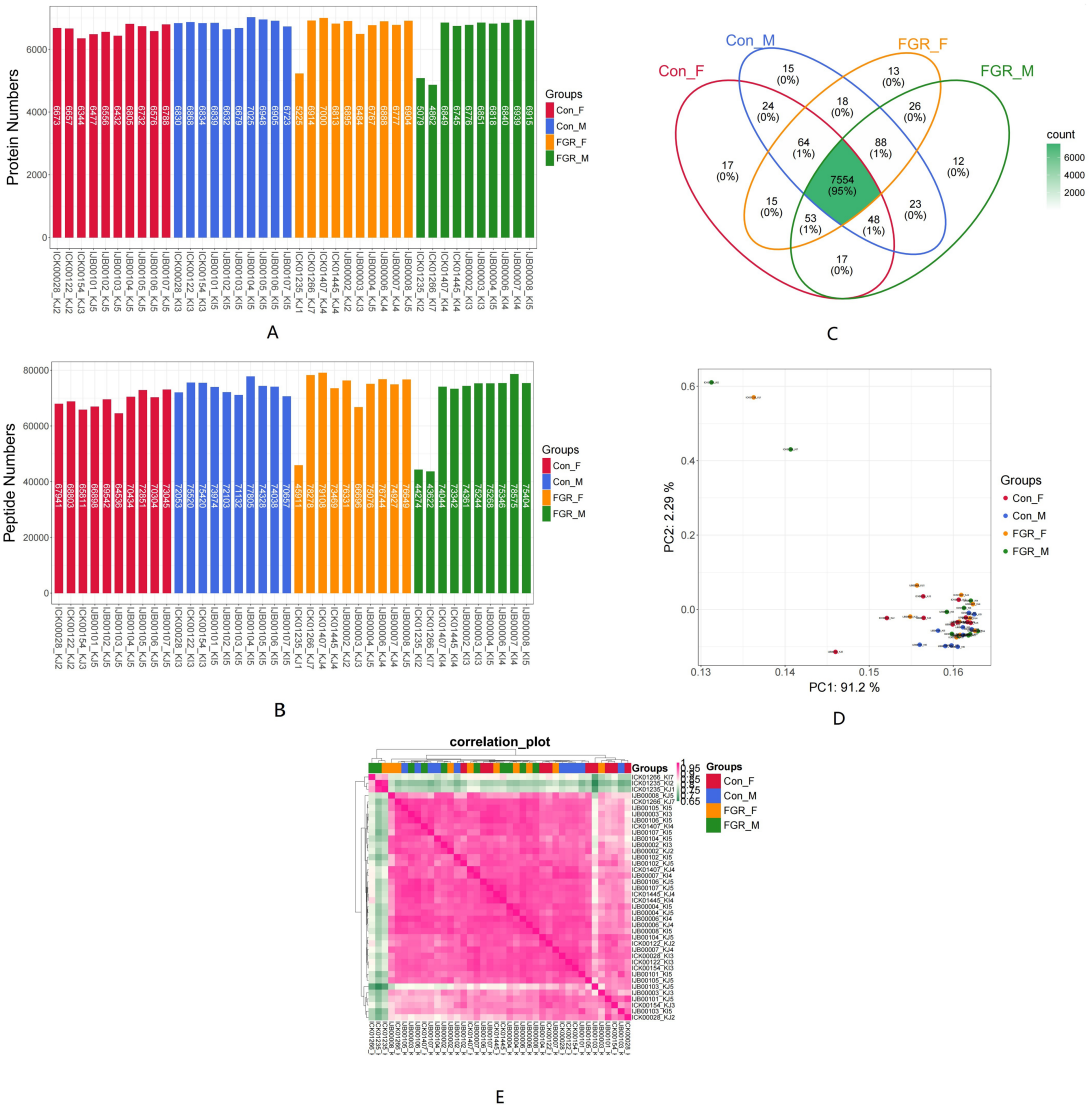
Quality control overview of the proteomic data from four placental sample groups: Con-F (fetal side of control placentas), Con-M (maternal side of control placentas), FGR_F (fetal side of FGR placentas), FGR-M (maternal side of FGR placentas). **(A)** Box-plot showing the total number of detected proteins across all individual samples. **(B)** Box-plot displaying the total number of detected peptide segments in each individual samples. **(C)** Venn diagram comparison to analyze and present the protein detection situation of each group. **(D)** PCA plot based on the quantitative profiles of all identified proteins. There are two outlier samples in the FGR-F group and one outlier in the FGR-M group was identified. **(E)** Pearson correlation coefficient analysis shows that the vast majority of specimens exhibited strong inter-sample correlation, except for three low-correlation samples.

Placental samples were categorized into maternal- and fetal-side, based on sampling location. The maternal side is the tissue adjacent to the uterine myometrium, and the fetal side is that adjacent to the fetal amniotic sac. All placental samples were divided into four group: fetal side of FGR (FGR-F), maternal side of FGR (FGR-M), fetal side of control (CON-F) and maternal side of control (CON-M). Before analyzing, we initiated a quality control assessment with box plots about the quantity of protein and peptide segments detected in each sample (Figures 2A,B). Subsequently, a Venn diagram comparison was employed to analyze and present the protein detection situation of each group (Figure 2C). Principal component analysis (PCA) performed on the identified DEPs showed that the group differences were not obvious, and there were two outlier samples in group FGR-F, and one outlier samples in group FGR-M (Figure 2D). Finally, Pearson correlation coefficient analysis was carried out across all placental samples (Figure 2E), demonstrating a strong overall correlation, except for three sample (the same sample as outlier in PCA analysis). consequently, these three outlier samples were removed from subsequent analysis.

### Identification of DEPs

To identified significant DEPs across groups, further differential screening was performed after 4D-DIA proteomic analyses. The screening criteria included the expression fold log2FC ≥ 1 or ≤ − 1 and FDR value (False Discovery Rate) ≤ 0.05, which was calculate using a T-test to assess statistical significance.

**Figure 3:**
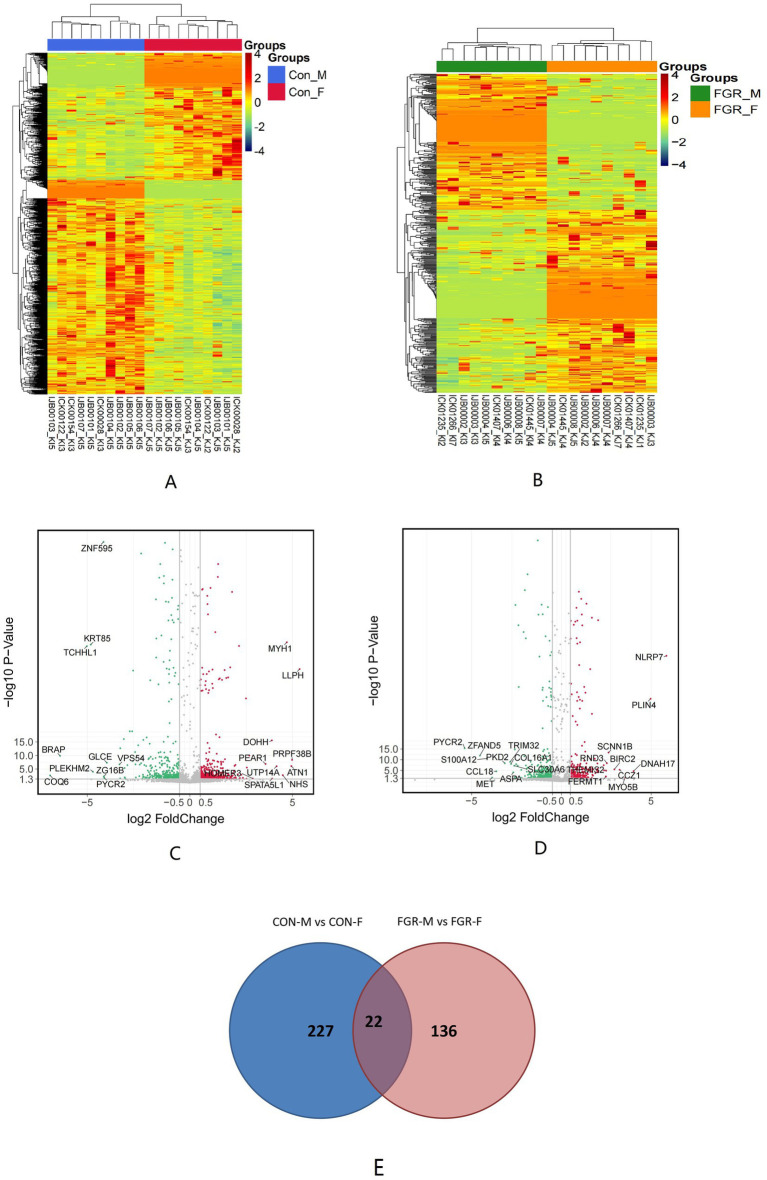
The DEPs between maternal and fetal side of placental in different group. **(A)** Hierarchical clustering heatmap showing the DEPs between CON-M and Con-F. **(B)** Hierarchical clustering heatmap showing the DEPs between FGR-M and FGR-F. **(C)** Volcano graphs of DEPs comparing the CON-M and CON-F. **(D)** Volcano graphs of DEPs comparing the FGR-M and FGR-F. **(E)** Venn diagram of DEPs.

First we compared the DEPs between the maternal and fetal sides with in the same group. There are 249 DEPs between CON-M and CON-F, including 114 upreguated and 135 downregulated proteins. In contrast, only 158 DEPs were identified between FGR-M and FGR-F, consisting of 76 proteins upregulated and 82 proteins downregulated proteins. Moreover only 22 DEPs were detected in both CON-M vs. CON-F and FGR-M vs. FGR-F comparisons. These finding indicate that the difference of protein expression between maternal and fetal side of placental in FGR group is less than in the normal group (Table 2; Figure 3).

**Table 2:** The DEPs in different groups.

Group	Number of DEPs	Upregulation	Downregulation
CON-M vs. CON-F	249	114	135
FGR-M vs. FGR-F	158	76	82
CON-F vs. FGR-F	278	154	124
CON-M vs. FGR-M	157	79	78

CON-M, maternal side of placental in control group; CON-F, fetal side of placental in control group; FGR-M, maternal side of placental in FGR group; FGR-F, fetal side of placental in FGR group.

**Figure 4:**
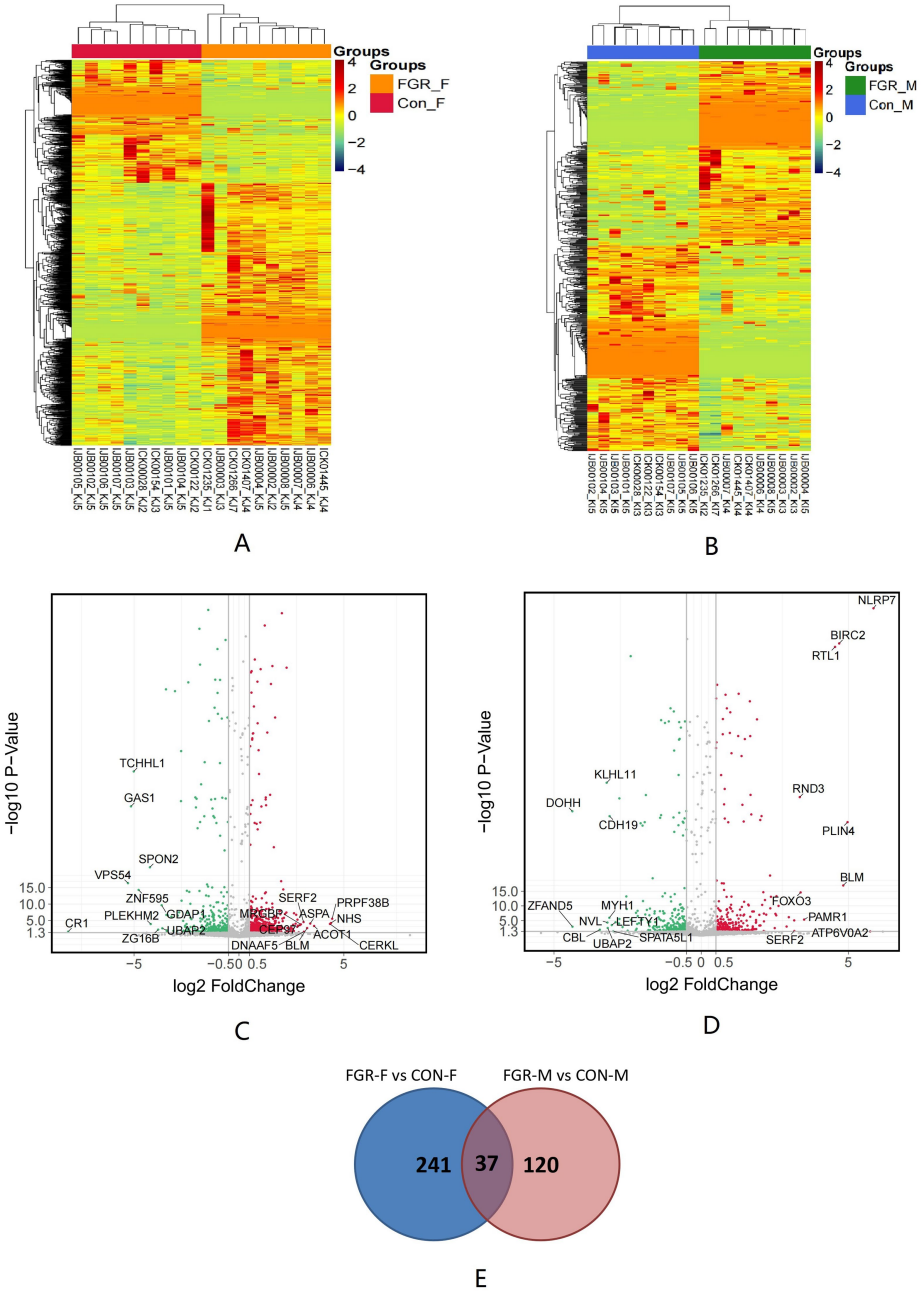
The DEPs between the same sides of placental in different group. **(A)** Hierarchical clustering heatmap showing the DEPs between FGR-G and Con-F. **(B)** Hierarchical clustering heatmap showing the DEPs between FGR-M and CON-M. **(C)** Volcano graphs of DEPs comparing the FGR-F and CON-F. Representative top 10 DEP gene names are labeled on the plot. **(D)** Volcano graphs of DEPs comparing the FGR-M and CON-M. Representative top 10 DEP gene names are labeled on the plot. **(E)** Venn diagram showing overlapping DEPs between Con-M vs. Con-F (227 unique DEPs) and FGR-M vs. FGR-F (136 unique DEPs), with only 22 shared DEPs across the two maternal-fetal pairwise comparisons, revealing reduced fetal-maternal side proteomic variation in FGR placentas.

Then we compared the same side in different groups. Between FGR-F and CON-F, we identified 278 DEPs (154 upregulated and 124 downregulated proteins). When compare FGR-M and CON-M, a total 157 DEPs (79 upregulated and 78 downregulated proteins) were detected. Only 37 DEPs both in FGR-F vs. CON-F and FGR-M vs. CON-M (Table 2; Figure 4). So the reduction in the difference in protein expression between the maternal and fetal surfaces of the placenta in the FGR group, primarily due to the abnormal expression of proteins on the fetal side.

### Functional analysis of DEPs

Go functional enrichment and enriched KEFF pathways were used to investigate the biological roles of the 278 DEPs identified in the CON-M vs. FGR-F comparison and 157 DEPs in the CON-M vs. FGR-M group comparison.

**Figure 5:**
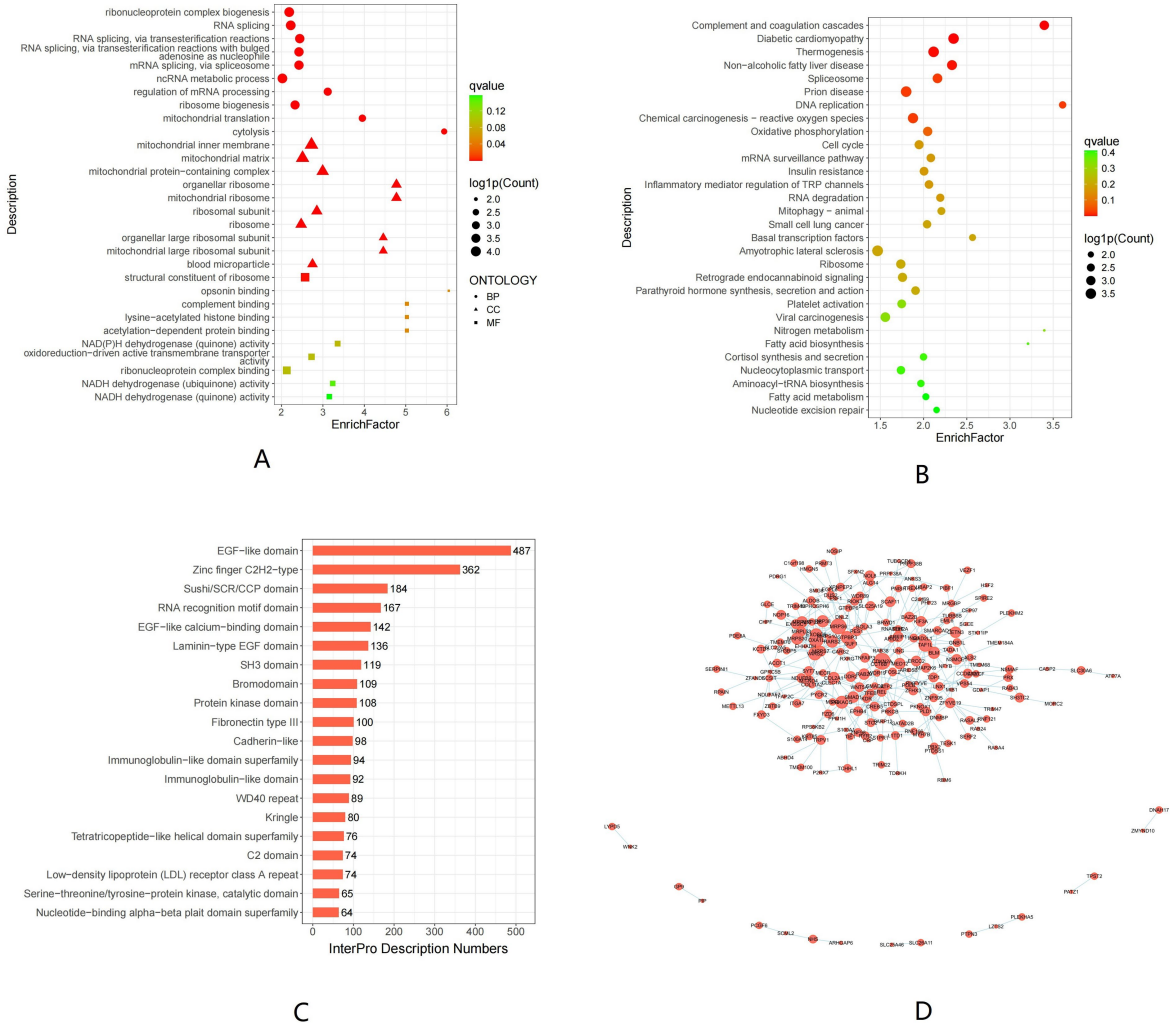
Bioinformatics analysis of DEPs from CON-F vs. FGR-F. **(A)** Go term enrichment statistics scatterplot chat (top 10). **(B)** Enrichment statistical scatterplot map of the KEGG pathway (top 20). **(C)** Bar plot of enrichment statistics of protein domain classification (top 20). **(D)** Diagram of protein–protein interaction networks shows that CDKN2A, MED12, ERCC2 in fetal side of placental were in the center of the network module and highly connected to other proteins.

The result from Go functional enrichment analysis about CON-F vs. FGR-F showed that: in biological processes (BP), DEPs were mainly enriched in ribonucleoprotein complex biogenesis, RNA slicing, RNA splicing, via transesterification reactions, RNA splicing, via transesterification reactions with bulged adenosine as nucleophile, mRNA splicing, via spliceosome. In the cellular component (CC) category, significant enrichments were observed in mitochondrial inner membrane, mitochondrial matrix, mitochondrial protein-containing complex, organellar ribosome, mitochondrial ribosome, ribosomal subunit. In the molecular function (MF) category, the most significance enriched item is opsonin binding, but both the adjusted *p* value and q value were greater than 0.05 (Figure 5A). The enriched KEGG pathways were complement and coagulation cascades, Diabetic cardiomyopathy, Thermogenesis, Non-alcoholic fatty liver disease, spliceosome, DNA replication, chemical carcinogenesis-reactive oxygen species (Figure 5B). The top 5 enriched protein structural domains included EGF-like domain, Zinc finger C2H2-type, Sushi/SCR/CCP domain, EGF-like calcium-binding domain, fibronectin type III (Figure 5C).

**Figure 6:**
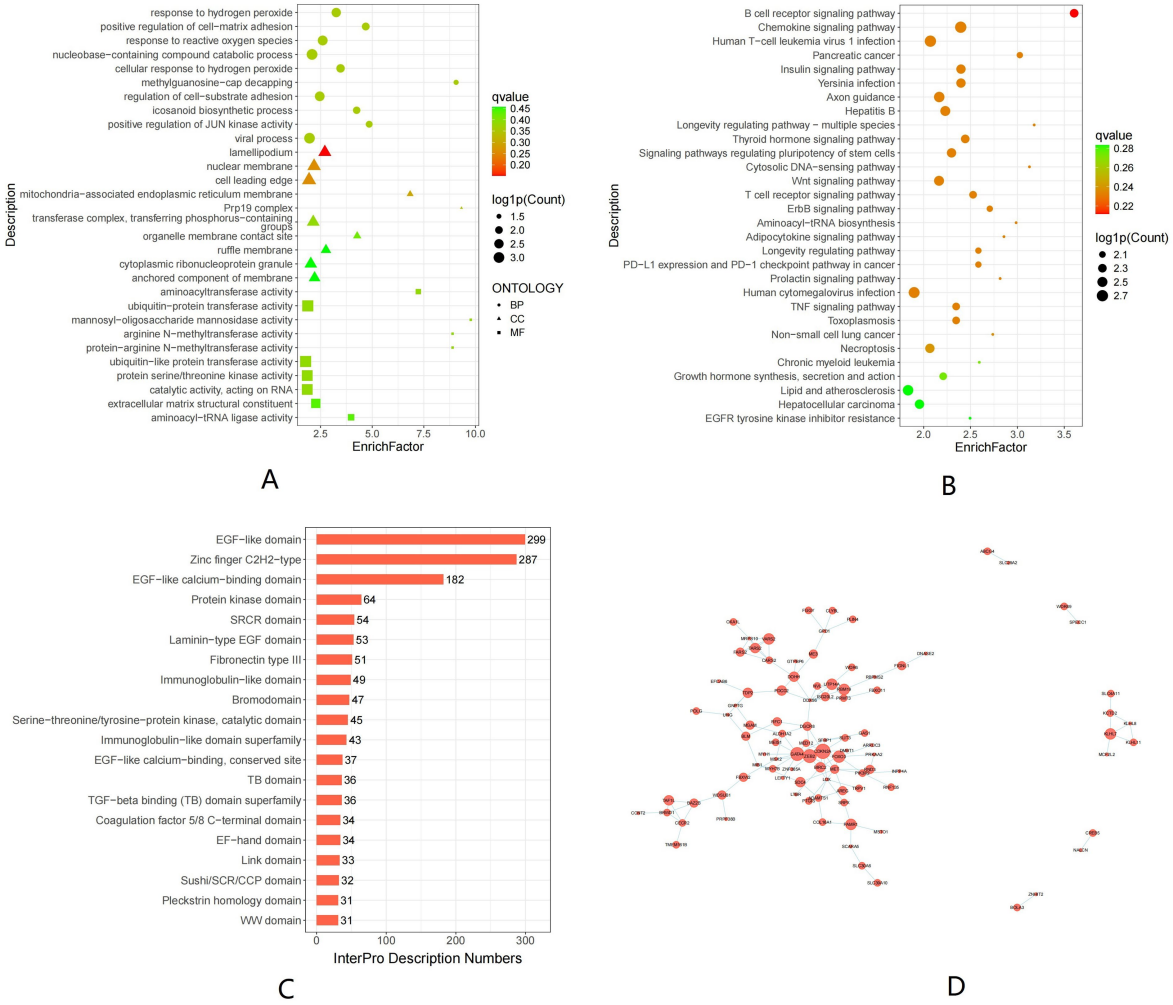
Bioinformatics analysis of DEPs from CON-M vs. FGR-M. **(A)** Go term enrichment statistics scatterplot chat (top 10). **(B)** Enrichment statistical scatterplot map of the KEGG pathway (top 20). **(C)** Bar plot of enrichment statistics of protein domain classification (top 20). **(D)** Diagram of protein–protein interaction networks show that GATA4, ZEB2, CDKN2A, FOXO3 in the mater side of placental were in the center of the network module and highly connected to other proteins.

In analysis the EDPS in FGR-M vs. CON-M, although some Go functional enrichment terms showed statistically significant *p*-value (*p* < 0.05), their adjusted *p* value and q value both above 0.05 (Figure 6A). A similar situation were observed in the KEGG pathway analysis (Figure 6B). The top 5 enriched protein structural domains included EGF-like domain, Zinc finger C2H2-type, EGF-like calcium-binding domain, protein-kinase domain and SRCR domain (Figure 6C).

### Protein-protein interaction networks (PPI)

**Figure 7:**
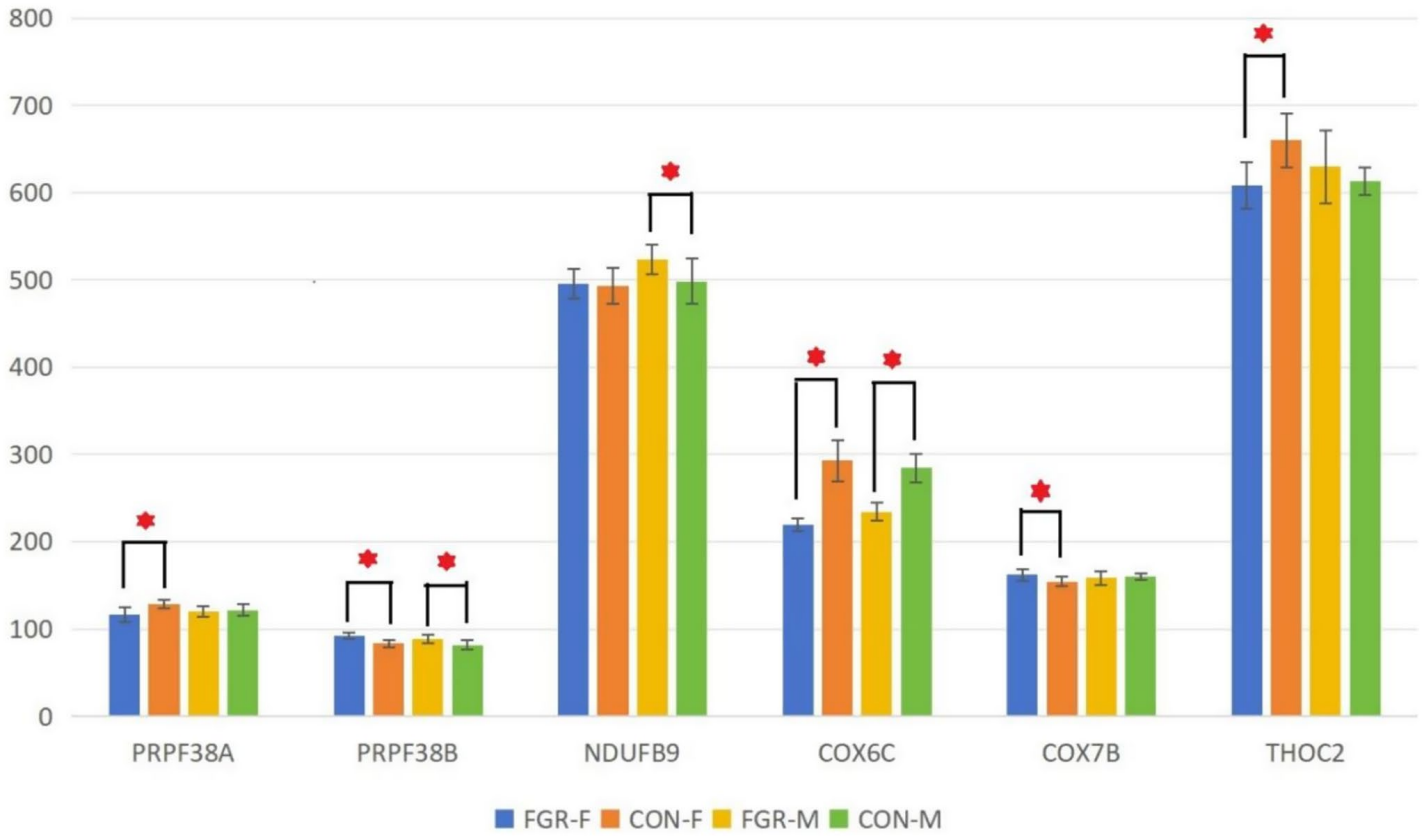
The ELISA results of the target protein in fetal and maternal sides of FGR group and control group. It shows that showed that PRPF38A, COX6C, THOC2 were down-regulated and PRPF38B and COX7B were up-regulated in the fetal side of the placenta in FGR group compared with the fetal side of control group. *p < 0.05.

In this study we selected 278 proteins form the CON-F vs. FGR-F comparison and 157 protein from the COM-M vs. FGR-M comparison for interaction network analysis. Through the PPI network analysis of the DEPs, CDKN2A (Cyclin-dependent kinase inhibitor 2A), MED12 (Mediator of RNA polymerase II transcription subunit 12), ERCC2 (TFIIH basal transcription factor complex helicase XPD subunit) et al. in fetal side of placental and GATA4 (Transcription factor GATA-4), ZEB2 (Zinc finger E-box-binding homeobox 2), CDKN2A, FOXO3 (Forkhead box protein O3) in the mater side of placental were in the center of the network module and highly connected to other proteins (Figure 7).

### Targeted validation by ELISA

For the main difference is focus on the fetal side of placental between the FGR group and the Control group, we selected the target proteins from the DEPs in the CON-F vs. FGR-F comparison. The DEPs main enriched in the following three items: mRNA splicing, via spliceosome; DNA replication preinitiation complex; and regulation of oxidative phosphorylation, as revealed by both Go functional enrichment analysis and KEFF pathways analyses. Target proteins were screened from the three items using the thresholds of FDR < 0.05 and|logFC| > 1.

Ultimately, the following proteins were identified as potential targets which may play a crucial role in the occurrence and/or development of FGR: PRPF38A, PRPF38B and TOCH2 from mRNA splicing via spliceosome item; NDUFB9, COX6C and COX7B from oxidative phosphorylation item. The ELISA results showed that PRPF38A, COX6C, THOC2 were down-regulated and PRPF38B and COX7B were up-regulated in the fetal side of the placenta in FGR group compared with the fetal side of control group, which were consistent with the proteomic analysis results. But NDUFB9 show no significant difference between FGR-F vs. CON-F comparison (Table 3; Figure 7).

**Table 3:** The ELISA results of the target protein in fetal and maternal sides of FGR group and control group.

Name	Group	*n*	Mean ± SD	*p*		*n*	Mean ± SD	*p*
PRPF38A	FGR-F	8	116.77 ± 8.26	0.002	FGR-M	8	120.61 ± 6.04	0.714
	CON-F	10	128.13 ± 4.76		CON-M	10	121.73 ± 6.60	
PRPF38B	FGR-F	8	92.26 ± 3.37	0.000	FGR-M	8	88.46 ± 4.81	0.014
	CON-F	10	83.28 ± 4.38		CON-M	10	81.76 ± 5.31	
NDUFB9	FGR-F	8	495.64 ± 16.66	0.770	FGR-M	8	523,06 ± 16.85	0.035
	CON-F	10	493.18 ± 20.81		CON-M	10	498.40 ± 26.13	
COX6C	FGR-F	8	219.24 ± 7.61	0.000	FGR-M	8	234.38 ± 10.07	0.000
	CON-F	10	292.86 ± 23.72		CON-M	10	284.38 ± 16.50	
COX7B	FGR-F	8	161.90 ± 6.66	0.017	FGR-M	8	158.36 ± 7.66	0.644
	CON-F	10	154.32 ± 5.40		CON-M	10	159.66 ± 3.85	
THOC2	FGR-F	8	608.59 ± 26.59	0.002	FGR-M	8	629.41 ± 41.77	0.267
	CON-F	10	659.91 ± 30.73		CON-M	10	612.99 ± 15.89	

For gestational age has a certain influence on the composition of placental tissue. In our research the average gestational age in FGR group is significantly smaller than control group, although both of them more than 35 gestational weeks. To minimize the influence of gestational age on the findings, we conducted a further subgroup analysis. All participants with FGR were divided into two groups according to gestational age at delivery. Those with gestational age at delivery greater than 37 weeks (total of 4 cases) were assigned to Group 2, and those with gestational age at delivery less than 37 weeks (including 2 cases at 33 weeks and 2 cases at 34 weeks of gestation) were assigned to Group 2. All participants in the normal control group were classified as Group 3.

As shown in Table 4, there were no statistically significant differences in the placental tissue of the levels of PRPF38A, PRPF38B, COX6C, COX7B and THOC2 between Group1 and Group2, all the participants in the two groups belong to FGR. However, statistically significant differences were observed when comparing these factors between the group1 vs. group3, and group2 vs. group3. This indicates that the placental expression levels of these five factors between FGR group and normal group are not associated with the gestational age, further suggesting that they play important roles in the occurrence and development of FGR.

**Table 4:** The relationship of the target proteins in different gestational weeks.

Name	Group			*p*		
	Group 1	Group 2	Group3	1 vs. 2	1 vs. 3	2 vs. 3
Number	4	4	10			
Gestational age	37.00 ± 8.16	33.50 ± 5.78	38.00 ± 8.16	0.00	0.81	0.02
PRPF38A	112.62 ± 9.62	120.92 ± 4.58	128.13 ± 4.76	0.17	0.01	0.00
PRPF38B	91.92 ± 4.41	92.59 ± 2.62	83.28 ± 4.38	0.80	0.00	0.00
NDUFB9	485.24 ± 22.67	501.13 ± 18.10	493.18 ± 20.81	0.32	0.29	0.52
COX6C	218.62 ± 7.62	219.86 ± 8.73	292.86 ± 23.72	0.84	0.00	0.00
COX7B	162.01 ± 8.40	161.80 ± 5.75	154.32 ± 5.40	0.97	0.06	0.04
THOC2	607.64 ± 38.88	609.55 ± 11.65	659.91 ± 30.73	0.93	0.02	0.00

## Discussion

FGR is defined as the fetus failing to reach its genetically predetermined growth potential. As known, fetal growth is influenced by a variety of factors, including uteroplacental function, maternal disease, maternal cardiovascular function, maternal nutrition and so on. Among these, uteroplacental insufficiency or dysfunction is one of the most common causes of FGR (11). Maternal vascular malperfusion, which arise from placental hypoplasia, infarcts and other lesions, results in failure of extravillous cytotrophoblast invasion, ultimately leading to poor placental development and inadequate remodeling of spiral arteries. Numerous studied have shown that FGR placentas exhibit reduced vascular branching in both the chorionic plate and capillary loops, and increased abundance of infarcts impairing blood of the villi (4, 12, 13). Along with the decrease in vascular density, FGR placental was often found increased incidence of fibrosis (14).

So in this research, we utilized 4D-DIA to investigate the different protein expression in placenta in both fetal and maternal side from FGR patients and normal pregnant women. The results showed that, compared to the control group the DEPs’ number between the maternal and fetal side was significantly reduced in the FGR group, means that the protein expression tended to be consistent on the two placenta side in FGR group. Further analysis showed that the consistent was primarily driven by changes on the fetal side, as the number of DEPs between FGR and normal group was greater on the fetal side than on the maternal side. So, we speculate that in normal pregnancies, the fetal side and the maternal side of the placenta have different functions, resulting in significant differences in their protein expression. However, in fetuses with FGR, the differences in protein expression between the maternal and fetal sides of the placenta diminish, leading to partial functional deficiencies. Moreover, the reduction of this difference may be mainly due to the alteration in protein expression on the fetal side, because the number of DEPs in the fetal side between the control group and the FGR group is larger than that in the maternal side between the two groups. Further analysis showed that PRPF38A, PRPF38B, and THOC2 (involved in mRNA splicing via spliceosome), COX6C and COX7B (associated with oxidative phosphorylation) may play a crucial role in the occurrence and/or development of FGR.

As early as 2014, Zhijing M et al. used tandem mass tags to construct a large-scale comparative proteomic profile of human placentas from normal and FGR pregnancies. They found 95 differentially expressed proteins between two groups, which were associated with two primary molecular networks related to erythropoiesis and oxidative stress (15). Geca T et al. used chromatographic mass spectrometry to compare the placental proteome profiles between pregnancies affected by late-onset FGR (EFW < 10th percentile, diagnosed after 32 weeks of pregnancy) and those with normal pregnancies (16). They identified 356 different proteins involved in regulating gene transcription, inhibiting proteolytic enzymes, controlling trophoblast proliferation and angiogenesis, and mediating inflammatory responses. These two studies share certain similarities with ours, especially the second one, which also found that proteins involved in regulating gene transcription may play important role in FGR. The discrepancies in research results are primarily attributed to differences in specimen selection criteria and the protein microarray detection methods adopted.

Marta et al. have demonstrated that the placenta possesses a distinct transcriptional landscape (17, 18). Perturbations in gene expression or post-translational modifications are likely to disrupt multiple essential physiological functions of the placenta. The further study indicated that aberrant splicing events lead to the generation of abnormal protein products, which further exert a pivotal effect on the pathogenesis of FGR (19). In our study we found that the expression levels of PRPF38A, PRPF38B and THOC2 which associated with mRNA splicing via spliceosome is significantly different in the fetal side between FGR and control group.

PRPF38A (pre-mRNA processing factor 38A), localized in nucleoplasm, is a component of the U2-type precatalytic splicesome complex, which contains a multi-interface protein–protein interaction domain, enables RNA binding activity (20, 21), and playing a role in RNA synthesis and processing (22). Little is known about PRPF38A or its encoded protein. Study by Diana L. C. et al. showed that knockdown of PRPF38A induced a dramatic morphological change in both hMSC-derived osteoblasts and might favor adipogenic differentiation to promote adipogenesis (23). Stefanie Ch et al. reported that PRPF38A proteins was positive related with the viability in Basal-A triple negative breast cancer cell lines (24). So, increase expressing of PAPF38A may promote cell proliferation and invasion. In our research, PAPF38A expression is down-regulated in placental from FGR patient, which may lead a decrease in the viability of trophoblast cells and/or other stromal cells on the fetal surface of placenta by aberrant splicing, potentially contributing to placental insufficiency and development of FGR.

PRPF38B (Pre-mRNA processing factor 38B), commonly perceived as being located in the nucleus, is considered to be part of precatalytic spliceosome, participating in mRNA splicing via spliceosome and enable RNA binding activity. As a unique component of the U4/U6. U5 tri small nuclear ribonuclear protein (snRNP) complex, PRPF38B plays a crucial role during spliceosome maturation. PRPF38B was first reported in human sarcoma and believed to be exclusively localized in the nucleus (25). Studies have shown that PRPF38B expression was negatively correlated with overall survival of ovarian cancer, breast cancer and gastric cancer patients through alternative splicing events (26–28). In our research, the expression of PAPF38B was significant higher in the fetal side of placental in FGR patients compared to that in normal placental. We hypothesize that up-regulation of PRPF38B may alter certain splicing events, leading to disordered proliferation of trophoblast cells and/or other stromal cells (e.g., irregular cell arrangement, imbalanced cell ratios, or increased numbers of non-function cells), ultimately resulting in placental dysfunction.

THOC2 is known as a component of the TREX complex involved in mRNA export from the nucleus to the cytoplasm (29), and plays a critical role in various cellular processes, including transcription, splicing and mRNA stability. THOC2 exhibits high expression levels in a variety of malignant tumors and knowing down THOC2 expression can markedly suppress tumor growth (30, 31). In our research, the level THOC2 is significantly lower in FGR group than normal control group. So we hypothesize that THOC2 may contribute to placental insufficiency and the occurrence of FGR by regulating the proliferation and function of trophoblast cells and/or vascular endothelial cells, possibly through modulating mRNA export and/or alternative splicing.

As known, mitochondria are critical organelles that control a variety of cellular processes, such as energy generation via oxidative phosphorylation, redox equilibrium maintenance, calcium signaling and apoptotic pathway regulation. Placental mitochondrial malfunction was a common observation in pregnancies complicated by FGR. It includes a complex interaction of reduced mitochondrial biogenesis, faulty oxidative phosphorylation, excessive oxidative stress and so on (32). In our study we found that the levels of COX6C and COX7B which associated with oxidative phosphorylation were significantly different in the fetal side of FGR group compared to the same side of control group, especially for COX6C is significantly down-regulated in both fetal and maternal side of FGR groups compared to the control group.

COX6C and COX7B are both subunits of cytochrome c oxidase, which is the terminal enzyme of the mitochondrial respiratory chain, catalyzes the electron transfer from reduced cytochrome c to oxygen. Both the two proteins are located in mitochondrial and participate in oxidative phosphorylation. Although the precise mechanisms by which the two protein contribute to the happen and/or development of FGR remain unclear, numerous studies have linked placental mitochondrial dysfunction with adverse perinatal outcomes including FGR or preeclampsia. ATP generation is significantly decreased in the placenta from FGR patients, indicating defective oxidative phosphorylation. This bioenergetic failure is likely caused by a combination of mtDNA depletion and direct damage to mitochondrial respiratory chain complex (33).

In conclusion, in this research we found that: (1). the number of DEPs between maternal and fetal sides of placental was significantly lower in the FGR group compared to the control group, indicating that the protein expression in both sides tends to become similar in FGR cases. (2) the number of DEPs between FGR and control group is greater on the fetal side than on the maternal side, indicating that the fetal side of placental may be the main contributor to placental dysfunction; (3) PRPF38A, PRPF38B, THOC2, COX6C and COX7B may play a critical roles in the onset and/or progression of FGR, suggesting that mitochondrial dysfunction and mRNA splicing abnormal in the placental are likely play important role in the pathophysiology of FGR.

So our studies may overturns the traditional research perspective that regards the placenta as a unified whole and verifies that the fetal side rather than the maternal side dominates the pathological progression of FGR. The homogenization of protein expression between maternal and fetal sides may be not a passive adaptive change of the placenta, but an adverse pathological outcome of fetal-side trophoblast injury. PRPF38A/PRPF38B/THOC2 mediates aberrant mRNA splicing and COX6C and COX7B mediates mitochondrial respiratory chain dysfunction in the fetal-side FGR placental trophoblast cells will be the core focus of our follow-up research.

### Limitation

Our study has some limitation. First, the sample size is small, for we only have 10 FGR and 10 control for 4D-DIA test, and for targeted DEPs validation by ELISA, we still only have 8 FGR and 10 control specimens. Second, as known, gestational age has a certain influence on the composition of placental tissue. In our research the average gestational age in FGR group is significantly smaller than control group. To exclude the influence of gestational age on the research results, the FGR group was further subdivided base on whether gestational age at delivery was greater than 37 weeks for the analysis of ELISA data. In addition to the statistical comparison within the FGR subgroups, comparisons were also performed between each FGR subgroup and the normal control group, respectively. The results demonstrated that there were no statistically significant differences in the target protein levels between the two FGR subgroups, whereas both subgroups exhibited statistically significant differences compared with the control group. These findings further validate the important roles of the target proteins in the occurrence and development of FGR. Third the experiments (both *in vivo* and *in vitro*) are needed to elucidate the biological functions of these selected factors.

## Data Availability

The datasets presented in this study can be found in online repositories. The names of the repository/repositories and accession number(s) can be found in the article/supplementary material. The data that support the findings of thsi study will be available in https://data.mendeley.com/datasets/hcrxt5zcgd/1, with the DOI: 10.17632/hcrxt5zcgd.1.
